# Association between multisite musculoskeletal pain and disability trajectories among community-dwelling older adults

**DOI:** 10.1007/s40520-024-02764-0

**Published:** 2024-05-23

**Authors:** Peiyuan Liu, Hongbo Chen, Beibei Tong, Disha Zhu, Xiaomei Cong, Shaomei Shang

**Affiliations:** 1 School of Nursing, Peking University, 38 Xueyuan Road, Haidian District, Beijing, 100191 China; 2 Nursing Department, Peking University Third Hospital, 49 North Garden Road, Haidian District, Beijing, 100191 China; 3 School of Nursing, Yale University, 400 West Campus Drive, Orange, Connecticut 06477 USA

**Keywords:** Multisite musculoskeletal pain, Disability, Activities of daily living, Group-based trajectory models, Older adults

## Abstract

**Background:**

Pain is linked to disability, but how multisite musculoskeletal pain leads to disability over time is not well elaborated.

**Objective:**

To examine the associations of multisite musculoskeletal pain with disability among a nationally representative cohort.

**Design:**

We used data from the National Health and Aging Trends Study (NHATS) 2015-22. Disability was assessed by basic activities of daily living (ADL) and instrumental activities of daily living (IADL).

**Participants:**

A total of 5557 individuals with multisite musculoskeletal pain dwelling in the community were included in this study.

**Methods:**

Group-based trajectory models were applied to identify distinct profiles of disability in ADL and IADL. Design-based logistic regressions were used to examine associations among multisite musculoskeletal pain, disability, and dual trajectory group memberships, adjusted for sociodemographic, health status, behavioral, and mental characteristics.

**Results:**

Persons who experienced multisite musculoskeletal pain were at higher risk of disability in ADL and IADL. We identified five heterogeneous disability trajectories and named them based on baseline levels and rates of increase over time. Approximately, 52.42% of older adults with multisite musculoskeletal pain were in trajectories with ADL and IADL declines, and 33.60% experienced a rapid decline. Multisite musculoskeletal pain was associated with elevated relative risk for the adverse disability trajectories, which generally increases with multisite musculoskeletal pain frequency and number of sites.

**Conclusions:**

Persons with multisite musculoskeletal pain had a higher risk of disability. It is essential to adopt effective pain management strategies to maintain the independent living ability of older adults and to realize active aging.

**Supplementary Information:**

The online version contains supplementary material available at 10.1007/s40520-024-02764-0.

## Key Points

• Persons with multisite musculoskeletal pain were at higher risk of disability.

• There is a dose-response correlation of disability with pain frequency and the number of sites.

• We studied a nationally representative sample of 5557 individuals with multisite musculoskeletal pain dwelling in the community.

**Supplementary Information:**

The online version contains supplementary material available at 10.1007/s40520-024-02764-0.

## Introduction

Musculoskeletal pain is highly prevalent among older adults in the community, with an incidence rate ranging from 27.6–33.6% [1]. Meanwhile, a considerable proportion of older adults experience high-impact pain, which refers to chronic pain that frequently restricts their daily life or work activities, with an incidence rate of 10.7%~15.8% [1]. Musculoskeletal disorders are associated with falls, frailty, mobility disorders, negative emotions, and dementia, which seriously affect the daily activities and well-being of older individuals [2–4]. Notably, research indicates that experiencing localized pain increases the risk of coexisting pain in other body areas, and that multisite pain has a worse prognosis, which may be related to a central sensitization of pain perception [5–7]. For instance, a study involving 7601 older adults revealed that approximately 39.6% reported being bothered by pain in multiple sites [8].

Despite the significant prevalence of multisite musculoskeletal pain among older adults, we are only now beginning to recognize its importance. Previous research has demonstrated that multisite musculoskeletal pain serves as a critical predictor of job disability among industrial workers after 4 years [9]. A 6-year cohort study from the Netherlands showed that people with multisite musculoskeletal pain had more often a lifetime depressive and/or anxiety disorder [10]. In addition, a study in China showed that multisite pain significantly increased the risk for dementia in adults aged 45 years or older [11]. However, the precise relationship between multisite musculoskeletal pain and daily functioning in older adults remains unclear.

Activities of daily living (ADL) refers to the daily repeated activities necessary for people to maintain the most basic survival and life, including self-care activities and functional mobility. Self-care activities included eating, grooming, washing, bathing, etc. Functional mobility included turning over, sitting up from bed, transferring, etc. Instrumental activities of daily living (IADL) refers to some activities that are necessary for people to maintain an independent life, including using the telephone, shopping, cooking, housework, etc. Previous studies have reported on the interrelationships between pain and ADL/IADL disability through cross-sectional analyses [12–14]. However, there is a lack of research examining the long-term trajectory of disability in older adults, particularly those with multisite musculoskeletal pain, over a follow-up period. By conducting long-term follow-up assessments, we can observe the declining pattern and trend of ADL in older adults, identify subgroups experiencing rapid decline, and analyze the associated factors. This research is of significant importance for preventing, predicting, and managing disability among older adults.

Furthermore, a strong dose-response relationship has been established between the number of multiple pain sites and disability, that is, the more pain sites, the higher the risk of disability [12, 15]. However, there is a dearth of studies investigating the relationship between the cumulative duration of multisite pain and disability. The associations between the number of pain sites and the duration of multisite pain with the longitudinal trajectory of disability remain unclear. In the current study, we hypothesized that multisite musculoskeletal pain contributes to ADL/IADL disability, and there is a dose-response correlation between the frequency of pain episodes and the number of sites with disability. To examine these hypotheses, we have used the National Health and Aging Trends Study (NHATS) to evaluate the association between multisite musculoskeletal pain and disability. Our study analyzes the cross-sectional relationship between pain and disability, explores the longitudinal trajectory of pain and disability, and investigates the relationship between the trajectory of disability and pain frequency as well as the number of pain sites.

## Methods

### Data sources and study population

The National Health and Aging Trends Study (NHATS) is a national longitudinal and prospective cohort study developed to represent individuals aged 65 and older who are Medicare beneficiaries and reside in the community, residential care, or nursing home in the United States. The data collection has been conducted annually since 2011 (Round 1) and has been replenished in 2015 (Round 5), with weighted response rates at each round ranging from 71.30 to 96.00%. To ensure diverse representation, individuals aged 90 and older and Black non-Hispanic individuals were oversampled using a stratified three-stage sample design. Regarding ethical considerations, the current study obtained an exemption from an institutional ethical review because it only utilized publicly available and de-identified data. Before participating in the study, informed consent was obtained from all participants [16].

**Fig. 1:**
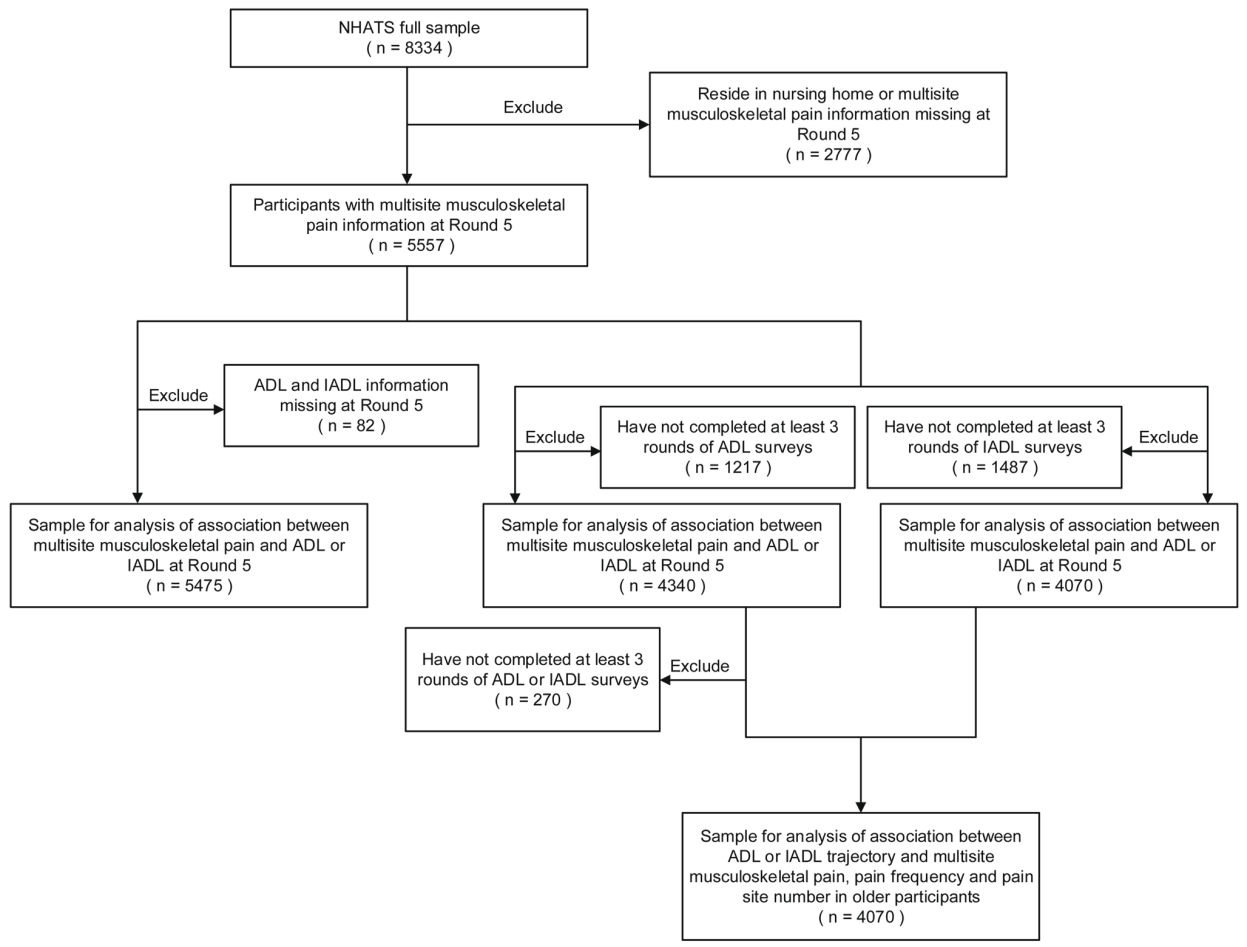
Flowchart of samples used for analyses

The current study used data from Round 5 to 11 of the NHATS which were collected from 2015 to 2022. The inclusion criteria of the participants incorporate: (1) individuals living in settings other than nursing homes to ensure the completion of a sample person questionnaire at the time of study enrollment; (2) participants with multisite musculoskeletal pain information in Round 5; (3) participants with ADL and IADL information, which requires completeness in the association analyses of multisite musculoskeletal pain and baseline ADL and IADL disability, or at least three rounds of ADL, IADL, or both assessment throughout the survey completed in the analyses of association between disability trajectories and multisite musculoskeletal pain. The specific sample inclusion process is shown in Fig. 1.

### Disability assessment

Disability was evaluated using two instruments. Disability in ADL was operationalized as a self- or proxy report of getting help from another person in the last month and having difficulty performing activities without help for each activity (eating, getting cleaned up, using the toilet, getting dressed, getting out of bed, and getting around inside the home) [17, 18]. Disability in IADL was defined as being in need of assistance on account of impairment in health or function and having difficulty with the following activities: laundry, shopping, making hot meals, managing finances, and keeping track of medicines [17]. Disability in ADL and IADL were dichotomized in the baseline association analysis, with non-zero values classified as having a disability. The number of disabilities in ADL and IADL were summed in the trajectory analyses.

### Multisite musculoskeletal pain assessment

At each interview, responders were asked: “In the last month, have you been bothered by pain?” and “Tell me where you had pain in the last month”. Based on the responses, we grouped participants into two groups: those who had more than one site of musculoskeletal pain (multisite), and those who had less than two sites (no multisite). We classified the number of pain sites as the largest number of musculoskeletal pain sites across the seven rounds, which included the back, hips, knees, feet, hands, wrists, shoulders, neck, arms, and legs. Pain frequency was defined as the number of rounds with multisite musculoskeletal pain.

### Covariates

**Sociodemographic variables** included age (measured in 10-year bands), gender, race/ethnicity (Non-Hispanic White, African American, Hispanic, other), education level (high school or less, some college or vocational school, bachelor or higher), annual income, marital status (married or partnered, single or widowed), enrollment in Medicare drug coverage (Part D), enrollment in Medicaid, and enrollment in Tricare.

**Health status parameters** incorporated body mass index (BMI), number of comorbidities, dementia status, and sensory impairment. BMI was calculated based on self-reported height and weight, categorized as underweight, normal, overweight, and obese. The number of comorbidities (0, 1–2, 3–8) was the total of the following chronic conditions based on self-reported doctor-diagnosis: heart attack, heart disease, hypertension, osteoporosis, diabetes, lung disease, stroke, and cancer, excluding arthritis for the high relevance of multisite musculoskeletal pain. Dementia status was defined as no dementia, possible dementia, or probable dementia based on a validated assessment method provided by NHATS [19]. Sensory impairment (none, single or dual) was assessed by self-reported difficulty with vision and hearing in certain situations. Individuals are considered to be vision impaired if they report blindness, inability to see well enough to recognize someone across the street, or to read newspaper print. Individuals are classified as hearing impaired if they have any of the following: deafness, hearing aid use, not being able to hear well enough to use the telephone, or to carry on a conversation in a room with a radio or TV playing [20].

**Behavioral and mental factors** consisted of social participation, depressive symptoms, and anxiety symptoms. Social participation was defined in alignment with a previously described typology as the total of the items, which are scored one point for reporting “Yes”, including living with at least one other person, talking to two or more people about “important matters” in the past year, attending religious services in the last month, and participating in organized activities (clubs, classes, or other) in the last month [21, 22]. Depressive symptoms and anxiety symptoms were obtained from the 2-item Patient Health Questionnaire-2 (PHQ-2) and 2-item Generalized Anxiety Disorder-2 scale (GAD-2), respectively, which both range from 0 to 6 and are dichotomized based on clinically significant cutoffs of 3 [23, 24].

### Statistical analysis

The complex survey design and sampling weight that adjusts for differential probabilities of selection and nonresponse in Round 5 were considered in the analysis, consequently permitting national estimates. In the first step, we carried out a design-based multivariate logistic regression analysis to identify the association between baseline disability and multisite musculoskeletal pain. Bivariate differences were compared using Rao-Scott Chi-square tests and design-based F-tests. In the next step, to better understand the impact of multisite musculoskeletal pain on disability, we applied group-based trajectory modeling (GBTM) through the traj-command in Stata, a specialized application of finite mixture modeling, using a zero-inflated Poisson distribution [25]. The best-fit model was selected based on Bayesian information and diagnostics including the average posterior probability (at least 0.7) and odds of correction classification (at least 5), as shown in E-methods of the Supplementary Data.

Thirdly, we conducted design-based multinomial logistic regression analyses to assess the association of multisite musculoskeletal pain frequency and the number of sites with disability trajectories. All analyses were performed using Stata/SE 17.0 (Stata Corp, College Station, TX) with a *P* value < 0.05 considered statistically significant.

## Results

### Association between baseline disability and multisite musculoskeletal pain

Baseline multisite musculoskeletal pain was associated with disability in ADL and IADL, and the relevance still existed after adjusting sociodemographic variables, health status parameters, and behavioral and mental factors by stage, as shown in Supplementary Fig. 1. In fully design-based logistic regression adjusted models, the relationship between multisite musculoskeletal pain and ADL and IADL disability was statistically significant (OR = 2.07 [95% CI: 1.76–2.44]; OR = 1.99 [95% CI: 1.69–2.34]).

### Identified disability trajectory groups

**Fig. 2:**
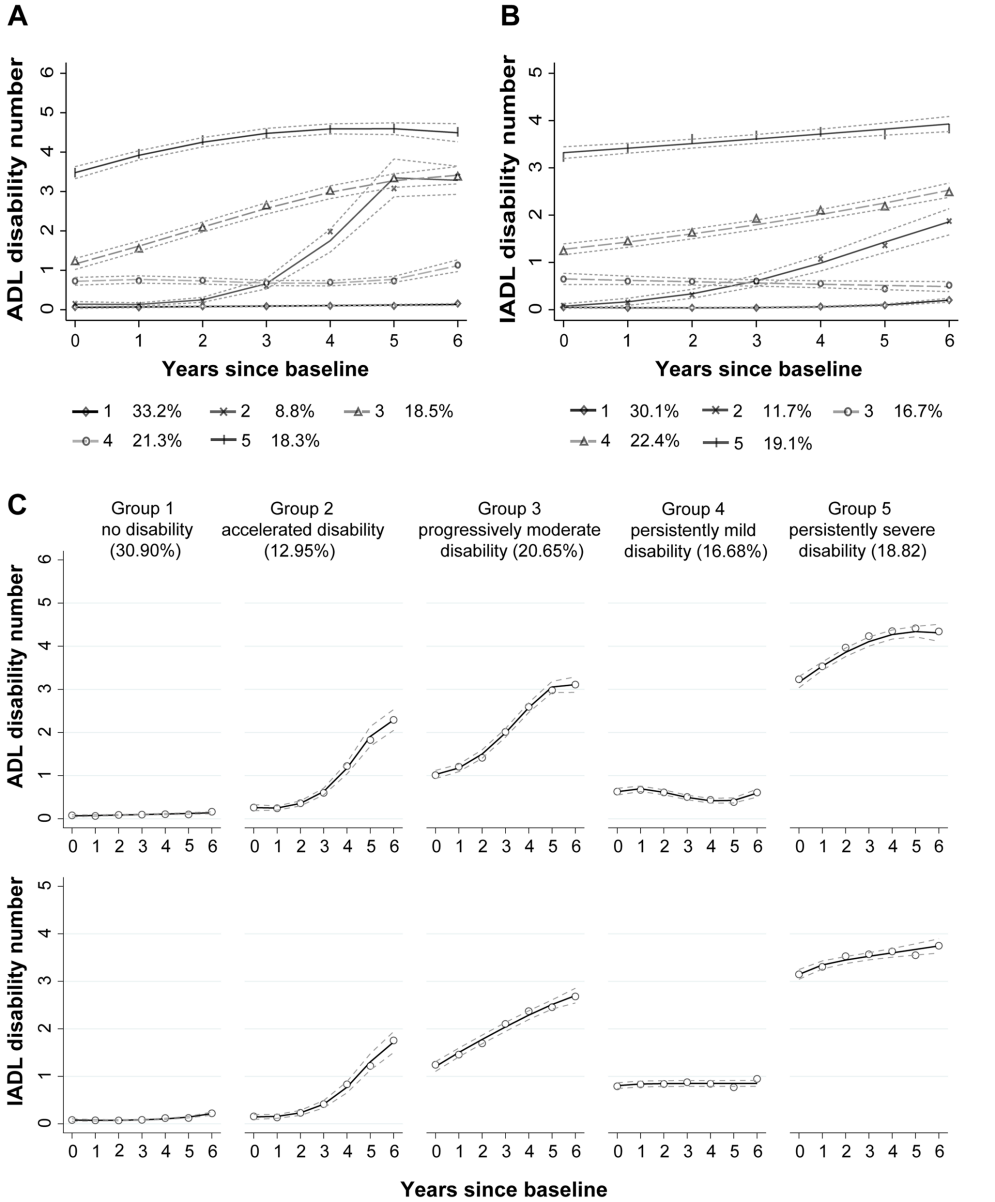
Estimated trajectory groups of disability among older adults

We categorized univariate trajectories and dual trajectories of disability in ADL and IADL (Fig. 2). Over seven years the frequency of disabilities in ADL and IADL increased among older adults, both those with multisite musculoskeletal pain and those without (Supplementary Figs. 1–3). We further identified five heterogeneous dual trajectory groups in the total participants, which are similar to subgroup analyses of individuals with/ without multisite musculoskeletal pain, and the estimated parameters are shown in Supplementary Tables 2–7. Based on the different baselines and various rates of increases over time, we labeled groups “no disability”, “accelerated disability”, “progressively moderate disability”, “persistently mild disability” and “persistently severe disability”, respectively. Approximately 30.90% of participants were in the “no disability” group with the lowest number of disabilities in ADL and IADL during the 7-round survey, 12.95% in the “accelerated disability” group with a baseline value close to 0, 20.65% in the “progressively moderate disability” group with a baseline value close to 1, 16.68% in the “persistently mild disability” group with a baseline value close to 1, and 18.82% in the “persistently severe disability” group with the highest baseline value close to 3.

### Differences in characteristics across subgroups

**Fig. 3:**
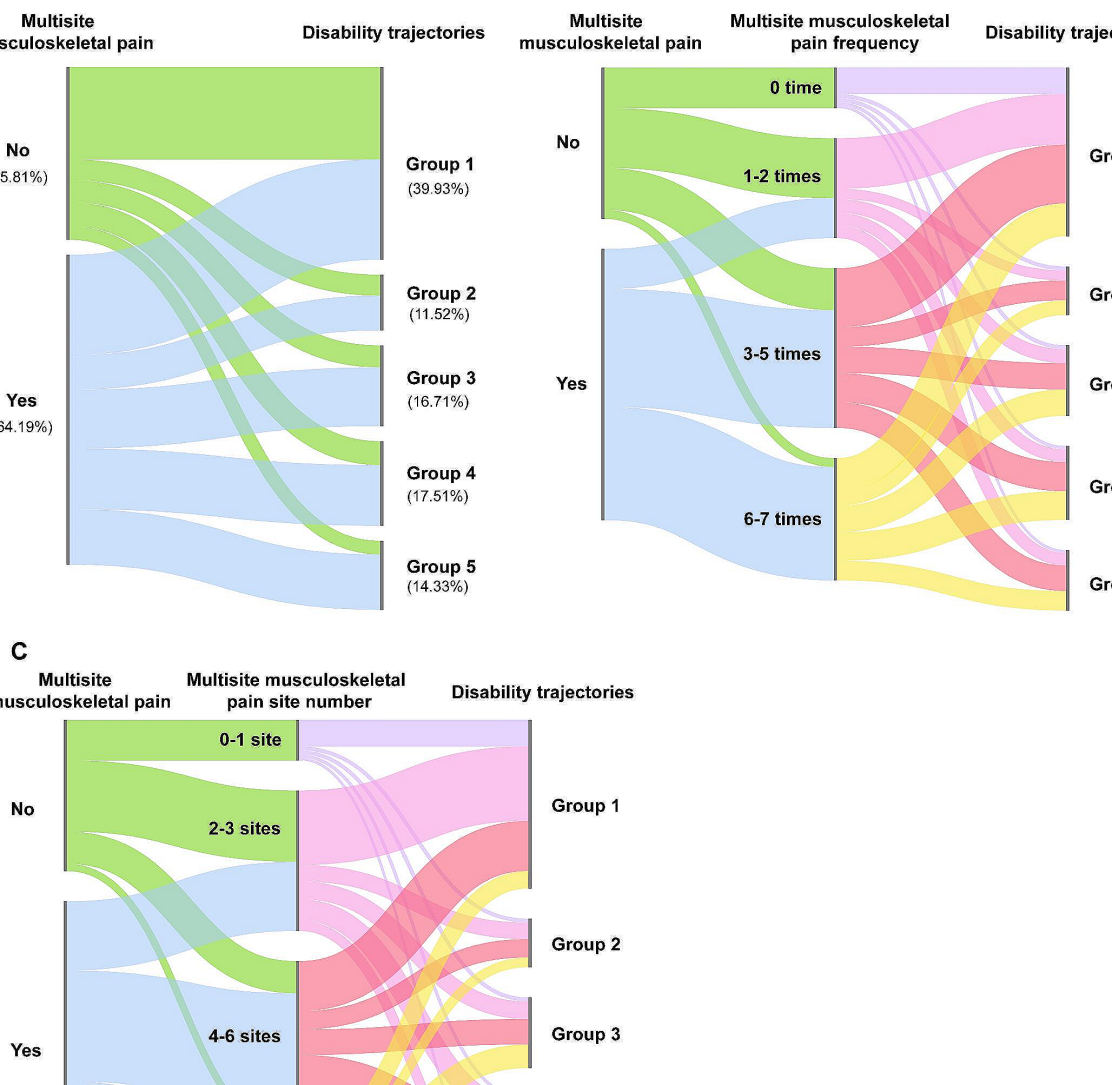
Sankey diagram (design adjusted proportion). The width of blocks on the left and medium correspond to group membership probabilities based on complex survey design. The width of blocks on the right corresponds to dual trajectory group membership probabilities. The width of the segments emanating out of each block corresponds to transition probabilities. **A** Group membership transitions in multisite musculoskeletal pain and disability trajectories. **B** Group membership transitions in multisite musculoskeletal pain, pain frequency, and disability trajectories. **C** Group membership transitions in multisite musculoskeletal pain, pain site number, and disability trajectories

**Fig. 4:**
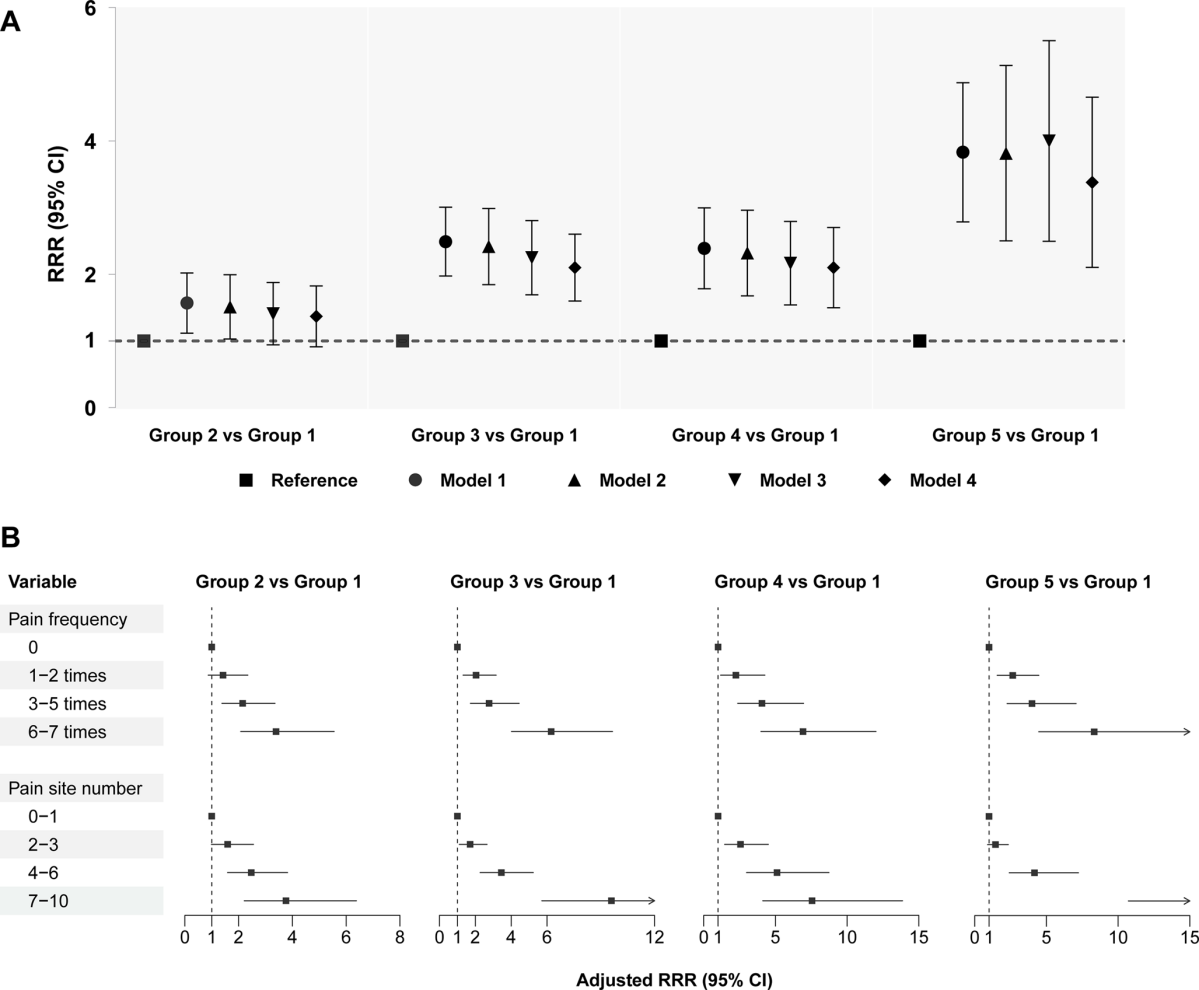
Association of disability trajectories with multisite musculoskeletal pain, pain frequency, and pain site number. **A** Correlates of multisite musculoskeletal pain and dual disability trajectories in multivariable analyses (model 1 was a crude model; model 2 adjusted for sociodemographic variables; model 3 additionally adjusted for health status parameters; model 4 additionally adjusted for behavioral and mental factors). **B** Pain frequency and number of pain sites as predictors of trajectory membership using multivariable multinomial logistic regression (model 4)

Baseline characteristics of the study population are shown in Supplementary Table 1. Table 1 compares sociodemographic, health status, and behavioral and mental outcomes across the 5 subgroups. Compared to Group 1 “no disability”, individuals in other groups were older, more likely to be female and non-White, had lower education levels and annual income, more likely to be single or widowed, have Medicare drug coverage and Medicaid, obese, have more comorbidities, probable dementia, single or dual sensory impairment, and have depressive and anxiety symptoms (*P* < 0.001). Figure 3A shows group membership transitions between multisite musculoskeletal pain and dual disability trajectories. The association between multisite musculoskeletal pain and disability trajectories is shown in Fig. 4A.

**Table 1:** Characteristics of study population across distinct disability subgroups (design adjusted mean or proportion)

Characteristics	Total	Group 1	Group 2	Group 3	Group 4	Group 5	*P* value
Age (y), %							< 0.001
65–74	59.09	73.44	51.73	41.77	62.02	41.62	
75–84	30.91	24.33	35.90	38.92	31.18	35.57	
≥ 85	10.00	2.23	12.37	19.31	6.80	22.81	
Female, %	59.38	55.66	50.20	63.12	65.52	65.26	< 0.001
Race/ethnicity, %							< 0.001
Non-Hispanic White	81.93	86.89	82.51	77.71	83.15	71.01	
African American	8.28	5.86	7.10	10.74	8.76	12.53	
Hispanic	6.59	4.33	8.11	7.41	5.18	12.43	
Other	3.21	2.92	2.28	4.14	2.91	4.04	
Education level, %							< 0.001
High school or less	41.47	32.41	45.14	49.13	36.81	60.77	
Some college or vocational school	29.71	32.19	27.99	26.23	34.59	22.21	
Bachelor or higher	28.83	35.40	26.87	24.63	28.61	17.02	
Annual income ($), %							< 0.001
< 15,000	14.79	6.50	13.27	22.39	13.39	31.98	
15,000–29,999	22.42	15.85	22.50	29.14	24.19	30.65	
30,000–44,999	15.20	14.73	18.44	16.25	15.90	11.86	
45,000–59,999	12.46	14.18	14.62	9.64	12.86	8.73	
≥ 60,000	35.13	48.75	31.17	22.57	33.66	16.78	
Marital status, %							< 0.001
Married or partnered	57.41	66.34	57.73	45.24	58.48	45.17	
Single or widowed	42.59	33.66	42.27	54.76	41.52	54.83	
Medicare drug coverage, %	65.98	61.04	66.72	68.66	66.44	75.97	< 0.001
Medicaid, %	11.96	5.18	10.97	16.25	8.81	30.99	< 0.001
Tricare, %	5.93	5.08	6.68	5.66	7.55	6.04	0.394
BMI, %							< 0.001
Underweight	1.13	0.73	0.27	1.80	0.55	2.95	
Normal	26.52	28.97	28.30	24.69	22.77	24.83	
Overweight	36.90	41.00	39.09	30.85	35.36	32.37	
Obese	35.46	29.30	32.34	42.66	41.32	39.85	
No. of comorbidities, %							< 0.001
0	14.41	21.72	12.61	6.86	12.33	6.82	
1–2	57.05	62.64	61.34	51.24	59.72	41.53	
3–8	28.54	15.64	26.05	41.90	27.94	51.64	
Dementia status, %							< 0.001
No dementia	87.84	96.67	91.06	82.59	92.90	60.59	
Possible	6.51	2.96	7.10	9.69	5.43	13.52	
Probable	5.65	0.37	1.85	7.72	1.67	25.89	
Sensory impairment, %							< 0.001
None	72.72	83.59	69.21	62.59	77.00	51.85	
Single or dual	27.28	16.41	30.79	37.41	23.00	48.15	
Social participation, mean (SE)	2.32 (1.00)	2.47 (0.89)	2.27 (1.07)	2.20 (1.08)	2.40 (0.94)	1.99 (1.09)	< 0.05; 1 and 4 > 3, 1 > 2 and 3 > 5
Depressive symptoms, %	12.62	4.84	10.20	18.56	11.24	30.97	< 0.001
Anxiety symptoms, %	10.69	4.66	7.80	13.47	9.58	27.92	< 0.001

National estimates based on complex survey design. SE, standard error. *P* value compared older adults in different subgroups

### Association of disability trajectories with multisite musculoskeletal pain frequency and number of pain sites

Figure 3B-C shows group membership transitions in multisite musculoskeletal pain, pain frequency or pain site number, and dual disability trajectories. Figure 4B shows the association between baseline disability and multisite musculoskeletal pain frequency. Compared with those who had no multisite musculoskeletal pain, the risk of Group 2 “accelerated disability”, Group 3 “progressively moderate disability”, Group 4 “persistently mild disability” and Group 5 “persistently severe disability”, and the risk generally increases with the pain frequency. The association between the number of multisite musculoskeletal pain sites and disability trajectories followed a similar interpretation to that of pain frequency.

## Discussion

In the population-based cohort study of older adults over the age of 65, we found that multisite musculoskeletal pain was associated with higher disability risk. We identified five distinct disability trajectories: “no disability” (30.90%), “accelerated disability” (12.95%), “progressively moderate disability” (20.65%), “persistently mild disability” (16.68%), and “persistently severe disability” (18.82%). In total, approximately 52.42% of older adults experienced ADL and IADL declines, and 33.60% experienced a rapid decline over seven years. Furthermore, the multinomial logistic regression results showed that compared with persons with no multisite musculoskeletal pain, those with multisite musculoskeletal pain had a higher risk of developing disability as the number of pain sites and frequency increased. The main results of this study are consistent with the previously proposed hypothesis.

First of all, for individuals experiencing functional decline, particularly those who exhibit rapid decline during the follow-up period, healthcare professionals need to promptly assess whether this decline can be reversed through early intervention. Timely intervention given within a reversible window period can significantly conserve medical care resources in the long term. Nevertheless, it is important to acknowledge that these trajectories also imply an increasing burden of disability care on healthcare systems and caregivers over time. Strategies need to be developed to address the public health burden of disability in older populations.

Second, previous research has primarily focused on describing the individual trajectory of either ADL or IADL in older adults [26–28]. In contrast, our study conducted dual trajectory analyses of both ADL and IADL. ADL and IADL represent fundamental daily life abilities and more complex instrumental life abilities, respectively. Existing research indicates that the prevalence of older adults with at least one ADL disability ranges from 5.05 to 9.77%, while the proportion of those with at least one IADL disability ranges from 6.01–14.85% [29]. Older adults often experience ADL and IADL disabilities through two pathways: (1) a catastrophic event, such as a hip fracture, or (2) a progressive decline in brain functions [30]. In our study, we identified functional decline from both ADL and IADL perspectives, particularly in populations with rapid decline. A comprehensive understanding of ADL and IADL disabilities among older individuals is crucial for society and healthcare institutions to develop targeted strategies. Healthcare professionals can employ tailored intervention strategies to delay the onset and/or progression of disability, such as fall prevention, early screening, and interventions for cognitive impairment and osteoporosis. By adopting such strategies, healthcare personnel can effectively address the challenges posed by ADL and IADL disabilities in the elderly population. At the same time, future research can use high-tech methods such as visual motion capture to develop objective assessment tools for living ability to increase the reliability and validity of the study.

Lastly, our findings underscore the association between multisite musculoskeletal pain and the risk of ADL and IADL disability in older adults, as evidenced by both baseline cross-sectional and longitudinal follow-up data. These associations were significantly observed even after adjusting for sociodemographic, health-related, behavioral, and mental related variables. Furthermore, our study revealed a dose-response relationship between the number of pain sites and the risk of disability, which is consistent with previous research [15, 31]. Additionally, we identified a novel dose-response relationship between the cumulative frequency of multisite pain and the risk of disability, which had not been demonstrated in previous studies.

Based on the results of this study, it is crucial to prioritize the management of multisite musculoskeletal pain. Effective control of the underlying conditions and primary diseases, judicious use of pain medications, and proactive use of physiotherapy methods such as photobiomodulation therapy, extracorporeal shock wave therapy, and acupuncture can prevent or reduce the occurrence of multisite pain [32–34]. These interventions have the potential to reduce the risk of disability in older adults, foster active aging, sustain independent living ability in the long term, and alleviate the burden on healthcare and social security systems. In addition, the development of multi-site pain prediction models for accurate prediction and management of pain is also a key strategy.

Pain can contribute to disability through multiple mechanisms. Pain increases the risk of falls by affecting sensorimotor function, with falls being the primary cause of nearly all hip fractures in older adults [35, 36]. Among hip fracture survivors, 50% lost functional independence, and one-third eventually became fully dependent [37]. Research has also demonstrated that persistent pain is associated with accelerated memory decline and an increased probability of dementia [2]. Chronic pain can directly or indirectly affect cognitive function through attention deficits, memory deficits, and emotional stress [38]. Furthermore, previous studies have explored the relationship between opioid drug use and central nervous system inhibition, suggesting that the abuse of painkillers may also be a risk factor for cognitive decline [39]. Lastly, in older adults, persistent pain might lead to the depletion of physiological reserves and impaired mobility, thereby increasing the risk of frailty [40]. Notably, frailty itself is an independent contributor to physical disability [41].

This study had notable strengths and limitations. A major strength was the utilization of a nationally representative dataset, which advances the generalizability of the findings to the entire older population in the United States. Additionally, the combination of cross-sectional and longitudinal analyses enabled us to draw clearer conclusions regarding the relationship between multisite musculoskeletal pain and ADL and IADL disability. However, it is important to acknowledge the presence of potential survival bias in our estimation results. This bias arises from the exclusion of individuals who are in poor health, leading to potentially optimistic estimates of the study results. In addition, the study did not incorporate objective measures of disability and pain, relying instead on self-reported or proxy-reported scales. Although this mode of assessment is widely recognized, it is important to consider its potential limitations. Future studies could use more objective outcomes to increase the credibility of the study. Finally, because only baseline data were applied for pain levels in this study, detailed analysis of the association between longitudinal changes in pain and disability trajectories was not possible.

Our findings have several important implications. First, the participants in the present study were drawn from the NHATS, a nationally representative database of older adults. As a result, our estimates can reflect the changing patterns of disability across the entire US older adult population. Specifically, our study identified two distinct patterns of functional changes among older adults: stable and declining trajectories. Additionally, within the group experiencing function decline, we identified subgroups with rapid decline. These trajectory estimates offer a clear understanding of the prevalence and long-term changes in disability among the elderly population in the United States.

In this study, we found that older adults with multisite musculoskeletal pain had a higher risk of disability compared to those without such pain. Furthermore, the risk of disability was observed to increase with the number of pain sites and the cumulative frequency of pain. From an interventional standpoint, implementing active and rational pain management strategies, including appropriate use of painkillers, effective management of primary diseases, and addressing the psychological and social well-being of older individuals, could effectively control multisite pain and delay the onset of disability. It is essential to conduct future studies aimed at identifying the risk factors contributing to the rapid disability subgroup among older adults. Such investigations would serve as a foundation for developing effective intervention strategies.

### Electronic supplementary material

Below is the link to the electronic supplementary material.

Supplementary Material 1

## Data Availability

No datasets were generated or analysed during the current study.
