# Supplementary material for: Association between multisite musculoskeletal pain and disability trajectories among community-dwelling older adults
Source: Aging Clin Exp Res. 2024 May 23;36(1):115. doi: 10.1007/s40520-024-02764-0 (PMC11116213; doi:10.1007/s40520-024-02764-0)
Supplement: Supplementary file 1 — Supplementary Material 1 [file 40520_2024_2764_MOESM1_ESM.docx]

**SUPPLEMENTARY DATA**

**Association Between Multisite Musculoskeletal Pain and Disability Trajectories among Community-Dwelling Older Adults**

**CONTENTS**

**E-methods.**

**Supplementary Table 1.** Baseline characteristics of the study population (design adjusted mean or proportion).

**Supplementary Table 2.** Model fit comparison for group-based trajectory models of total sample.

**Supplementary Table 3.** Group-based trajectory diagnostics for activities of daily living (ADL), instrumental activities of daily living (IADL) and dual models of total sample.

**Supplementary Table 4.** Model fit comparison for group-based trajectory models of persons with multisite musculoskeletal pain.

**Supplementary Table 5.** Group-based trajectory diagnostics for activities of daily living (ADL) and instrumental activities of daily living (IADL) models of persons with multisite musculoskeletal pain.

**Supplementary Table 6.** Model fit comparison for group-based trajectory models of persons without multisite musculoskeletal pain.

**Supplementary Table 7.** Group-based trajectory diagnostics for activities of daily living (ADL) and instrumental activities of daily living (IADL) models of persons without multisite musculoskeletal pain.

**Supplementary Table 8** Association between times of multi-site musculoskeletal pain and disability trajectories.

**Supplementary Table 9** Association between of multi-site musculoskeletal pain sites number and disability trajectories.

**Supplementary Fig. 1.** Association between multisite musculoskeletal pain and disability.

**Supplementary Fig. 2.** Estimated trajectory groups of disability among older adults with multisite musculoskeletal pain.

**Supplementary Fig. 3.** Estimated trajectory groups of disability among older adults without multisite musculoskeletal pain.

**E-methods.**

We identified distinct trajectories of disability in ADL and IADL using group-based trajectory modeling (GBTM), a data-driven method that can divide individuals with similar patterns over time into different groups using model fitting based on maximum likelihood estimation[1]. The outcome of disability in ADL and IADL were modeled using the continuous number of activities with difficulty or needed help (zero-inflated Poisson distribution) and time as years since enrollment. We hypothesized that disability in ADL and IADL would follow two to six distinct and clinically meaningful trajectories, respectively and jointly[2]. Thus, we fit models using the two-step process outlined by Nagin. During the first step, we modeled all trajectory groups with cubic polynomial terms, so differences in model fit parameters were only due to varying the number of trajectories. We chose the model with minimum Bayesian information criterion (BIC) values, minimum Akaike information criterion (AIC) values, average posterior probability (AvePP) of no less than 0.70, and minimum estimated group size of no less than 5%. Next, we fixed the number of trajectories identified in the previous step and ran the models with different potential combinations of polynomial forms (linear, quadratic, and cubic), identifying the optimal model using BIC values. We also assessed the group-based trajectory diagnostics, using statistically oriented criteria, as suggested by Nagin[1], including (a) obtaining for each trajectory group a close correspondence between the estimated probability of group membership and the proportion assigned to that group based on the posterior probability of group membership, (b) ensuring that the average of the posterior probabilities of group membership for individuals assigned to each group exceeds a minimum threshold of 0.7, (c) establishing that the odds of correct classification based on the posterior probabilities of group membership exceed a minimum threshold of 5, and (d) observing reasonably tight confidence intervals around estimated group membership probabilities.

**References**

1. Nagin DS, Odgers CL. Group-based trajectory modeling in clinical research. Annu Rev Clin Psychol. 2010;6:109-38.

2. Gill TM, Gahbauer EA, Han L, Allore HG. The role of intervening hospital admissions on trajectories of disability in the last year of life: prospective cohort study of older people. BMJ (Clinical research ed). 2015 May 20;350:h2361.

**Supplementary Table 1.** Baseline characteristics of the study population (design adjusted mean or proportion).

| Characteristics | Total  (N = 5475) | Persons with multisite musculoskeletal pain  (n = 3637) | Persons without multisite musculoskeletal pain  (n = 1838) | *P* value |
| --- | --- | --- | --- | --- |
| Age (y), % |  |  |  | 0.031 |
| 65-74 | 57.27 | 56.50 | 58.67 |  |
| 75-84 | 30.59 | 30.50 | 30.76 |  |
| ≥85 | 12.14 | 13.00 | 10.57 |  |
| Female, % | 59.32 | 61.51 | 55.33 | 0.002 |
| Race/ethnicity, % |  |  |  | 0.517 |
| Non-Hispanic White | 80.68 | 80.09 | 81.78 |  |
| African American | 8.51 | 8.96 | 7.69 |  |
| Hispanic | 6.92 | 7.17 | 6.46 |  |
| Other | 3.88 | 3.78 | 4.07 |  |
| Education level, % |  |  |  | < 0.001 |
| High school or less | 44.05 | 46.93 | 38.75 |  |
| Some college or vocational school | 29.27 | 29.19 | 29.41 |  |
| Bachelor or higher | 26.68 | 23.88 | 31.83 |  |
| Annual income ($), % |  |  |  | < 0.001 |
| <15000 | 16.02 | 18.74 | 11.09 |  |
| 15000-29999 | 23.51 | 25.60 | 19.70 |  |
| 30000-44999 | 15.47 | 15.11 | 16.13 |  |
| 45000-59999 | 12.44 | 11.92 | 13.39 |  |
| ≥60000 | 32.56 | 28.64 | 39.69 |  |
| Marital status, % |  |  |  | < 0.001 |
| Married or partnered | 56.36 | 53.21 | 62.08 |  |
| Single or widowed | 43.64 | 46.79 | 37.92 |  |
| Medicare drug coverage, % | 66.13 | 68.39 | 61.96 | 0.002 |
| Medicaid, % | 12.93 | 14.59 | 9.89 | < 0.001 |
| Tricare, % | 6.10 | 5.91 | 6.45 | 0.540 |
| BMI, % |  |  |  | < 0.001 |
| Underweight | 1.93 | 1.67 | 2.40 |  |
| Normal | 28.12 | 26.32 | 31.41 |  |
| Overweight | 35.65 | 34.57 | 37.63 |  |
| Obese | 34.30 | 37.44 | 28.55 |  |
| No. of comorbidities, % |  |  |  | < 0.001 |
| 0 | 13.27 | 10.86 | 17.66 |  |
| 1-2 | 56.14 | 54.01 | 60.03 |  |
| 3-8 | 30.58 | 35.13 | 22.30 |  |
| Dementia status, % |  |  |  | 0.063 |
| No dementia | 84.36 | 83.45 | 86.02 |  |
| Possible | 7.76 | 8.14 | 7.09 |  |
| Probable | 7.88 | 8.42 | 6.90 |  |
| Sensory impairment, % |  |  |  | < 0.001 |
| None | 71.36 | 68.88 | 75.89 |  |
| Single or dual | 28.64 | 31.12 | 24.11 |  |
| Social participation, mean (SE) | 2.24 (1.01) | 2.20 (1.02) | 2.32 (0.98) | < 0.05 |
| Depressive symptoms, % | 14.20 | 17.48 | 8.23 | < 0.001 |
| Anxiety symptoms, % | 12.68 | 15.90 | 6.82 | < 0.001 |

National estimates based on complex survey design. SE, standard error. *P* value compared older adults between persons with and without multisite musculoskeletal pain.

**Supplementary Table 2.** Model fit comparison for group-based trajectory models of total sample.

| Classes | Bayesian Information Criterion (BIC) | Akaike Information Criterion (AIC) | Average posterior probability (AvePP)^a^ | Estimated group size (%) |  |
| --- | --- | --- | --- | --- | --- |
| Activities of daily living trajectory groups (*N* = 4340) | | | | | |
| 2 | -36023.22 | -35982.34 | 0.97 | 45.35-54.65 |  |
| 3 | -34436.67 | -34497.99 | 0.90-0.95 | 28.10-39.75 |  |
| 4 | -34141.05 | -34059.29 | 0.81-0.94 | 18.29-33.34 |  |
| **5** | **-33890.47** | **-33788.27** | **0.80-0.91** | **9.26-33.69** |  |
| 6 | -33883.27 | -33760.63 | 0.68-0.90 | 1.16-33.38 |  |
| Instrumental activities of daily living trajectory groups (*N* = 4070) | | | | | |
| 2 | -30937.31 | -30896.82 | 0.97-0.98 | 43.20-56.80 |  |
| 3 | -29740.53 | -29679.78 | 0.89-0.94 | 26.44-40.90 |  |
| 4 | -29544.29 | -29463.30 | 0.81-0.91 | 19.60-29.68 |  |
| **5** | **-29447.50** | **-29346.26** | **0.70-0.90** | **12.00-30.11** |  |
| 6 | -29415.72 | -29294.23 | 0.65-0.90 | 7.34-28.51 |  |
| Dual trajectory groups (*N* = 4070) | | | | | |
| 2 | -64516.93 | -64433.20 | 0.98 | 44.70-55.30 |  |
| 3 | -61528.44 | -61405.04 | 0.94-0.97 | 27.51-39.77 |  |
| 4 | -60649.94 | -60486.88 | 0.90-0.95 | 19.91-29.85 |  |
| **5** | **-60188.33** | **-59985.61** | **0.83-0.94** | **12.63-31.34** |  |
| 6 | -60218.34 | -59975.96 | 0.83-0.93 | 0.20-31.21 |  |

a: Minimum threshold = 0.70

**Supplementary Table 3.** Group-based trajectory diagnostics for activities of daily living (ADL), instrumental activities of daily living (IADL) and dual models of total sample.

| Groups | Average posterior probability (AvePP)^a^ | Odds of correct classification (OCC)^b^ | Actual group size (%) | Estimated group size (%) |
| --- | --- | --- | --- | --- |
| Activities of daily living trajectory groups (*N* = 4340) | | | | |
| 1 | 0.90 | 43.00 | 35.35 | 33.17 |
| 2 | 0.80 | 85.37 | 7.63 | 8.78 |
| 3 | 0.80 | 37.84 | 18.64 | 18.52 |
| 4 | 0.81 | 35.02 | 20.35 | 21.27 |
| 5 | 0.90 | 87.57 | 18.04 | 18.27 |
| Instrumental activities of daily living trajectory groups (*N* = 4070) | | | | |
| 1 | 0.88 | 40.82 | 32.31 | 30.09 |
| 2 | 0.71 | 41.27 | 10.27 | 11.73 |
| 3 | 0.70 | 26.95 | 15.68 | 16.68 |
| 4 | 0.80 | 32.44 | 22.80 | 22.39 |
| 5 | 0.90 | 90.34 | 18.94 | 19.11 |
| Dual disability trajectory groups (*N* = 4070) | | | | |
| 1 | 0.93 | 79.97 | 31.74 | 30.90 |
| 2 | 0.83 | 72.80 | 12.16 | 12.95 |
| 3 | 0.89 | 70.62 | 20.81 | 20.65 |
| 4 | 0.83 | 55.27 | 16.49 | 16.68 |
| 5 | 0.95 | 176.91 | 18.80 | 18.82 |

a: Minimum threshold = 0.70

b: Minimum threshold = 5.00

**Supplementary Table 4.** Model fit comparison for group-based trajectory models of persons with multisite musculoskeletal pain.

| Classes | Bayesian Information Criterion (BIC) | Akaike Information Criterion (AIC) | Average posterior probability (AvePP)^a^ | Estimated group size (%) |  |
| --- | --- | --- | --- | --- | --- |
| Activities of daily living trajectory groups (*N* = 2879) | | | | | |
| 2 | -25594.56 | -25555.74 | 0.97 | 48.11-51.89 |  |
| 3 | -24608.23 | -24550.01 | 0.90-0.95 | 31.97-34.83 |  |
| 4 | -24393.17 | -24315.54 | 0.81-0.93 | 20.76-27.59 |  |
| **5^b^** | **-24234.85** | **-24137.81** | **0.79-0.90** | **10.31-28.07** |  |
| 6 | -24186.94 | -24070.50 | 0.74-0.90 | 7.35-27.15 |  |
| Instrumental activities of daily living trajectory groups (*N* = 2682) | | | | | |
| 2 | -21785.42 | -21747.01 | 0.97 | 46.95-53.05 |  |
| 3 | -21061.83 | -21004.21 | 0.88-0.95 | 31.90-35.65 |  |
| 4 | -20952.06 | -20875.24 | 0.80-0.90 | 23.64-27.36 |  |
| **5** | **-20895.80** | **-20799.76** | **0.71-0.90** | **11.66-24.93** |  |
| 6 | -20888.51 | -20773.27 | 0.69-0.89 | 6.81-24.27 |  |

a: Minimum threshold = 0.70

b: For activities of daily living trajectory groups, the optimal model was identified as five groups, although the model with six groups had minimum BIC values, due to the obvious overlapping confidence intervals (CIs).

**Supplementary Table 5.** Group-based trajectory diagnostics for activities of daily living (ADL) and instrumental activities of daily living (IADL) models of persons with multisite musculoskeletal pain.

| Groups | Average posterior probability (AvePP)^a^ | Odds of correct classification (OCC)^b^ | Actual group size (%) | Estimated group size (%) |
| --- | --- | --- | --- | --- |
| Activities of daily living trajectory groups (*N* = 2879) | | | | |
| 1 | 0.89 | 77.73 | 29.63 | 27.89 |
| 2 | 0.79 | 105.45 | 9.03 | 10.24 |
| 3 | 0.79 | 50.69 | 19.35 | 19.89 |
| 4 | 0.80 | 50.04 | 21.57 | 21.16 |
| 5 | 0.90 | 114.93 | 20.42 | 20.81 |
| Instrumental activities of daily living trajectory groups (*N* = 2682) | | | | |
| 1 | 0.87 | 74.75 | 26.73 | 24.91 |
| 2 | 0.71 | 65.71 | 10.07 | 11.21 |
| 3 | 0.71 | 43.46 | 15.88 | 16.93 |
| 4 | 0.79 | 44.69 | 23.94 | 23.55 |
| 5 | 0.90 | 105.37 | 23.38 | 23.41 |

OCC odds of correct classification

a: Minimum threshold = 0.70

b: Minimum threshold = 5.00

**Supplementary Table 6.** Model fit comparison for group-based trajectory models of persons without multisite musculoskeletal pain.

| Classes | Bayesian Information Criterion (BIC) | Akaike Information Criterion (AIC) | Average posterior probability (AvePP)^a^ | Estimated group size (%) |  |
| --- | --- | --- | --- | --- | --- |
| Activities of daily living trajectory groups (*N* = 1461) | | | | | |
| 2 | -10258.48 | -10223.01 | 0.97 | 38.36-61.64 |  |
| 3 | -9787.08 | -9733.88 | 0.89-0.95 | 19.44-50.61 |  |
| 4 | -9675.41 | -9604.48 | 0.82-0.93 | 14.88-44.26 |  |
| **5 ^b^** | **-9627.68** | **-9539.03** | **0.78-0.94** | **11.01-39.09** |  |
| 6 | -9595.96 | -9489.58 | 0.78-0.93 | 8.07-31.29 |  |
| Instrumental activities of daily living trajectory groups (*N* = 1388) | | | | | |
| 2 | -9039.47 | -9004.36 | 0.96-0.97 | 37.03-62.97 |  |
| 3 | -8633.29 | -8580.61 | 0.92-0.95 | 14.63-52.18 |  |
| 4 | -8570.02 | -8499.79 | 0.78-0.93 | 11.44-38.90 |  |
| **5** | **-8550.67** | **-8462.88** | **0.70-0.93** | **11.39-39.38** |  |

a: Minimum threshold = 0.70

b: For activities of daily living trajectory groups, the optimal model was identified as five groups, although the model with six groups had minimum BIC values, due to the obvious overlapping confidence intervals (CIs).

**Supplementary Table 7.** Group-based trajectory diagnostics for activities of daily living (ADL) and instrumental activities of daily living (IADL) models of persons without multisite musculoskeletal pain.

| Groups | Average posterior probability (AvePP)^a^ | Odds of correct classification (OCC)^b^ | Actual group size (%) | Estimated group size (%) |
| --- | --- | --- | --- | --- |
| Activities of daily living trajectory groups (*N* = 1461) | | | | |
| 1 | 0.81 | 121.22 | 18.89 | 19.82 |
| 2 | 0.92 | 135.21 | 47.36 | 45.22 |
| 3 | 0.82 | 185.42 | 13.62 | 13.76 |
| 4 | 0.82 | 311.10 | 7.26 | 8.02 |
| 5 | 0.93 | 564.27 | 12.87 | 13.17 |
| Instrumental activities of daily living trajectory groups (*N* = 1388) | | | | |
| 1 | 0.70 | 111.21 | 10.01 | 12.20 |
| 2 | 0.88 | 104.78 | 40.85 | 38.40 |
| 3 | 0.68 | 79.91 | 14.99 | 15.90 |
| 4 | 0.83 | 125.54 | 22.69 | 21.83 |
| 5 | 0.93 | 652.12 | 11.46 | 11.66 |

OCC odds of correct classification

a: Minimum threshold = 0.70

b: Minimum threshold = 5.00

**Supplementary Table 8** Association between times of multi-site musculoskeletal pain and disability trajectories.

| Pain times^a^ | Group 2 vs Group1 | | Group 3 vs Group1 | | Group 4 vs Group1 | | Group 5 vs Group1 | |
| --- | --- | --- | --- | --- | --- | --- | --- | --- |
|  | RRR (95% CI) | *P* | RRR (95% CI) | *P* | RRR (95% CI) | *P* | RRR (95% CI) | *P* |
| 0 | 1.00 (ref) |  | 1.00 (ref) |  | 1.00 (ref) |  | 1.00 (ref) |  |
| 1-2 times | 1.42 (0.87, 2.34) | 0.161 | 2.04 (1.32, 3.16) | 0.002 | 2.24 (1.18, 4.26) | 0.015 | 2.65 (1.57, 4.47) | < 0.001 |
| 3-5 times | 2.15 (1.38, 3.35) | 0.001 | 2.77 (1.73, 4.44) | < 0.001 | 4.06 (2.37, 6.96) | < 0.001 | 4.00 (2.26, 7.07) | < 0.001 |
| 6-7 times | 3.39 (2.08, 5.55) | < 0.001 | 6.23 (4.02, 9.65) | < 0.001 | 6.92 (3.99, 12.01) | < 0.001 | 8.33 (4.47, 15.52) | < 0.001 |

RRR, relative risk ratio; 95% CI, 95% confidence interval.

a: Pain times were counted across seven waves of survey.

All effect estimates were adjusted for age, gender, race/ethnicity, education level, annual income, marital status, Medicare drug coverage, Medicaid, Tricare, BMI, number of comorbidities, dementia status, sensory impairment, social participation, depressive symptoms and anxiety symptoms.

**Supplementary Table 9** Association between of multi-site musculoskeletal pain sites number and disability trajectories.

| Pain sites number^a^ | Group 2 vs Group1 | | Group 3 vs Group1 | | Group 4 vs Group1 | | Group 5 vs Group1 | |
| --- | --- | --- | --- | --- | --- | --- | --- | --- |
|  | RRR (95% CI) | *P* | RRR (95% CI) | *P* | RRR (95% CI) | *P* | RRR (95% CI) | *P* |
| 0-1 | 1.00 (ref) |  | 1.00 (ref) |  | 1.00 (ref) |  | 1.00 (ref) |  |
| 2-3 | 1.59 (0.99, 2.55) | 0.052 | 1.71 (1.11, 2.65) | 0.016 | 2.56 (1.46, 4.50) | 0.001 | 1.45 (0.90, 2.34) | 0.121 |
| 4-6 | 2.47 (1.59, 3.82) | < 0.001 | 3.45 (2.27, 5.23) | < 0.001 | 5.11 (2.99, 8.73) | < 0.001 | 4.17 (2.41, 7.23) | < 0.001 |
| 7-10 | 3.76 (2.21, 6.38) | < 0.001 | 9.59 (5.71, 16.10) | < 0.001 | 7.55 (4.12, 13.86) | < 0.001 | 21.09 (10.72, 41.50) | < 0.001 |

RRR, relative risk ratio; 95% CI, 95% confidence interval.

a: Pain sites number was the largest number of musculoskeletal pain sites across seven waves of survey.

All effect estimates were adjusted for age, gender, race/ethnicity, education level, annual income, marital status, Medicare drug coverage, Medicaid, Tricare, BMI, number of comorbidities, dementia status, sensory impairment, social participation, depressive symptoms and anxiety symptoms.


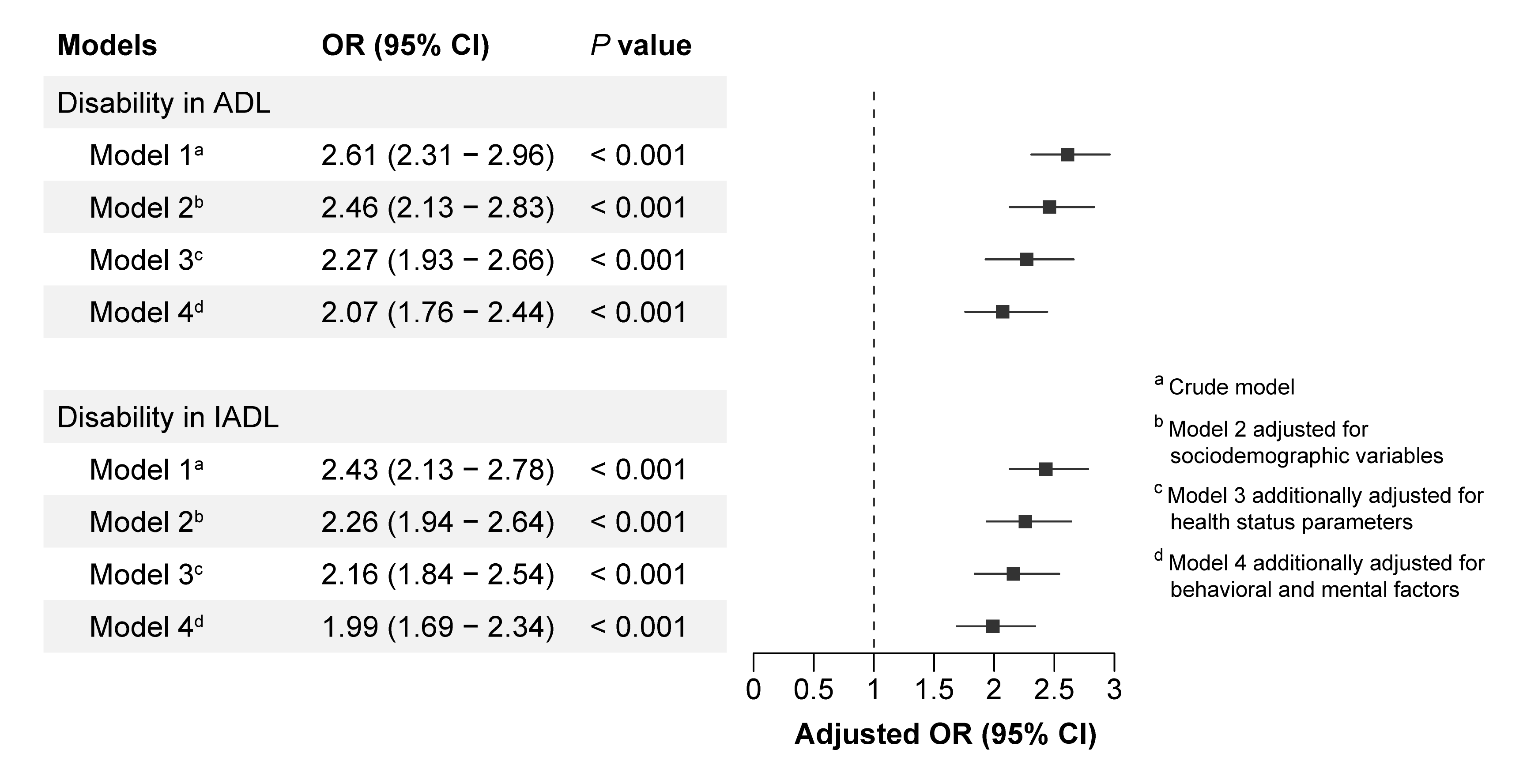


**Supplementary Fig. 1.** Association between multisite musculoskeletal pain and disability.

OR, odds ratio; 95% CI, 95% confidence interval.

ORs were calculated using multivariate logistic regression model.

a: Model 1 was a crude model including ADL and IADL disability and multisite musculoskeletal pain.

b: Model 2 adjusted for sociodemographic variables, including age, gender, race/ethnicity, education level, annual income, marital status, Medicare drug coverage, Medicaid and Tricare.

c: Model 3 additionally adjusted for health status parameters, including BMI, number of comorbidities, dementia status and sensory impairment.

d: Model 4 additionally adjusted for behavioral and mental factors, including social participation, depressive symptoms and anxiety symptoms.


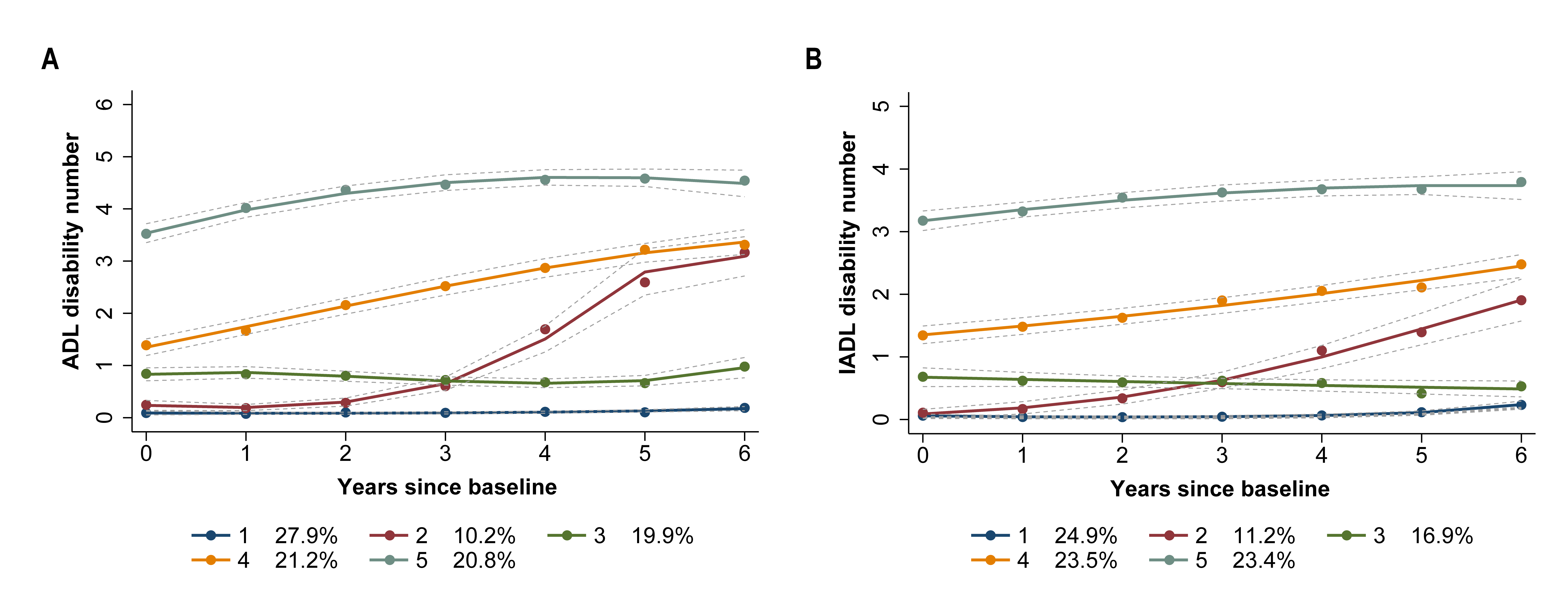


**Supplementary Fig. 2.** Estimated trajectory groups of disability among older adults with multisite musculoskeletal pain. **A** 5-group ADL trajectories. **B** 5-group IADL trajectories.


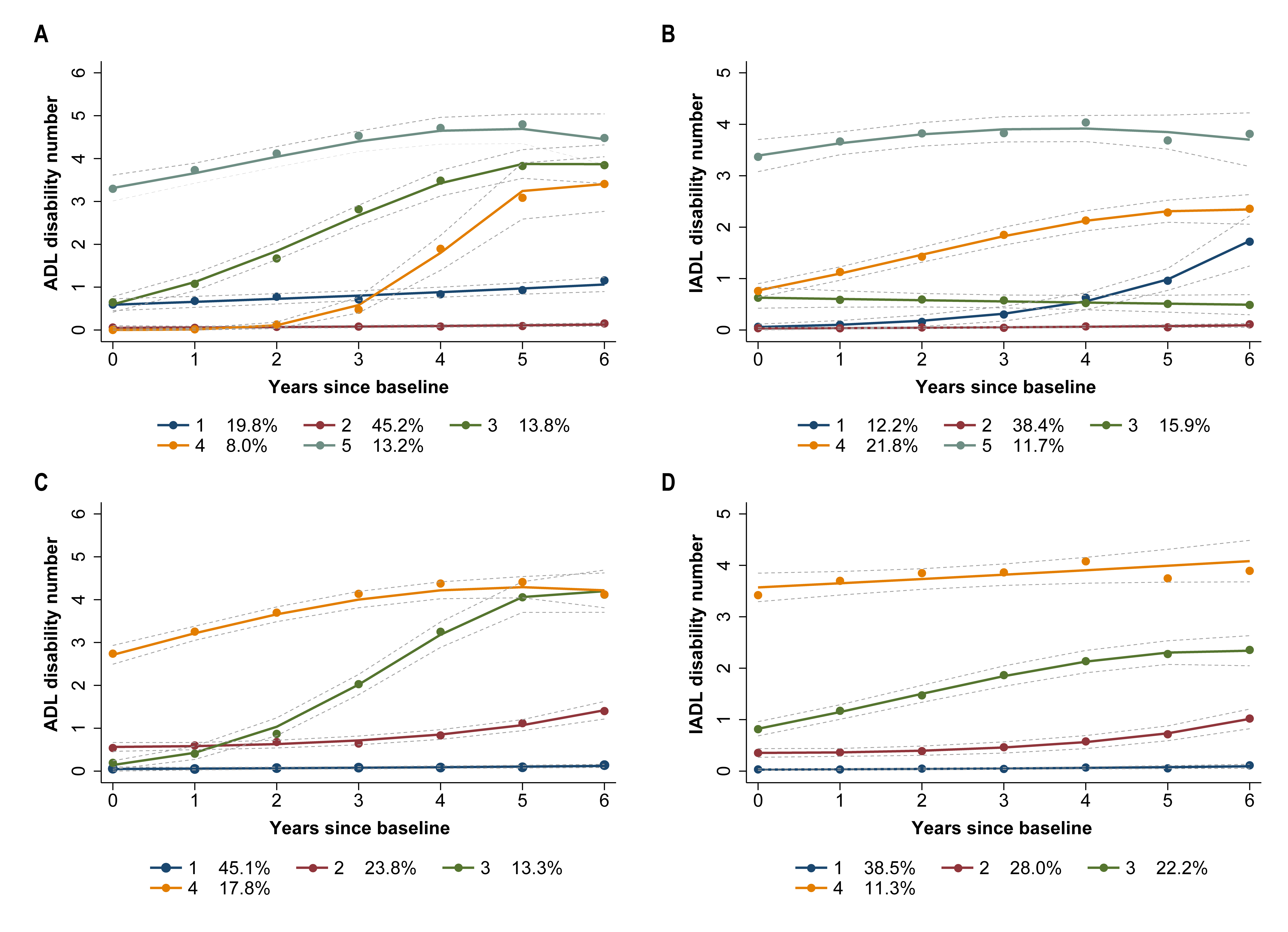


**Supplementary Fig. 3.** Estimated trajectory groups of disability among older adults without multisite musculoskeletal pain. **A** 5-group ADL trajectories. **B** 5-group IADL trajectories. **C** 4-group ADL trajectories. **D** 4-group IADL trajectories.
